# A Gradualist Scenario for Language Evolution: Precise Linguistic Reconstruction of Early Human (and Neandertal) Grammars

**DOI:** 10.3389/fpsyg.2016.01714

**Published:** 2016-11-08

**Authors:** Ljiljana Progovac

**Affiliations:** Linguistics Program, Wayne State UniversityDetroit, MI, USA

**Keywords:** language evolution, proto-grammar, syntactic reconstruction, “living fossils, ” insult, (pre-)adaptation, (sexual) selection, Neandertal language

## Abstract

In making an argument for the antiquity of language, based on comparative evidence, Dediu and Levinson (2013) express hope that some combinations of structural features will prove so conservative that they will allow deep linguistic reconstruction. I propose that the earliest stages of syntax/grammar as reconstructed in Progovac (2015a), based on a theoretical and data-driven linguistic analysis, provide just such a conservative platform, which would have been commanded also by Neandertals and the common ancestor. I provide a fragment of this proto-grammar, which includes flat verb-noun compounds used for naming and insult (e.g., *rattle-snake, cry-baby, scatter-brain*), and paratactic (loose) combinations of such flat structures (e.g., *Come one, come all; You seek, you find*). This flat, binary, paratactic platform is found in all languages, and can be shown to serve as foundation for any further structure building. However, given the degree and nature of variation across languages in elaborating syntax beyond this proto-stage, I propose that hierarchical syntax did not emerge once and uniformly in all its complexity, but rather multiple times, either within Africa, or after dispersion from Africa. If so, then, under the uniregional hypothesis, our common ancestor with Neandertals, *H. heidelbergensis*, could not have commanded hierarchical syntax, but “only” the proto-grammar. Linguistic reconstructions of this kind are necessary for formulating precise and testable hypotheses regarding language evolution. In addition to the hominin timeline, this reconstruction can also engage, and negotiate between, the fields of neuroscience and genetics, as I illustrate with one specific scenario involving FOXP2 gene.

## What can the comparative evidence from bones and genes tell us?

Based on the comparative evidence involving the descendants of *H. heidelbergensis* (*H. sapiens*, Denisovans, and Neandertals), Dediu and Levinson (2013) propose that at least *H. heidelbergensis* had some form of language. They reach this conclusion after reviewing a number of recent findings concerning genetics, skeletal morphology, the morphology of the vocal tract, infant maturation, brain size, and cultural artifacts. According to Dediu and Levinson (2013: 10), “language as we know it must then have originated within the ~1 million years between *H. erectus* and the common ancestor of Neandertals and us.” The authors conclude that Neandertals and Denisovans “had the basic genetic underpinning for recognizably modern language and speech, but it is possible that modern humans may outstrip them in some parameters (perhaps range of speech sounds or rapidity of speech, *complexity of syntax*, size of vocabularies, or the like)” (p. 5; emphasis mine).

Dediu and Levinson's (2013) proposal was completely dismissed by Berwick et al. (2013). Interestingly, however, Berwick and Chomsky (2016) in their latest work have (quietly) shifted their view on this. While they do not acknowledge this, they have significantly shifted their estimated date of the emergence of language to up to 200,000 years ago (e.g., p. 157), from the previous “just a bit over 50,000 years ago” (Chomsky, 2005; see also Berwick and Chomsky, 2011). In this respect, Berwick and Chomsky (2016) met almost half way Dediu and Levinson's (2013) estimate that language dates back to the common ancestor of humans and Neanderthals, to some 400,000–500,000 years ago. Not only that, but Berwick and Chomsky (2016) no longer claim that Neanderthals did not have language. Instead, they now say that it is the “$64,000 question whether Neandertals had language” (p. 50)^1^.

Relevant for these considerations are the recent findings suggesting that the derived FOXP2 variant that was initially thought to be uniquely human (Enard et al., 2002) is not entirely so, and that Neandertals also have a derived variant (Krause et al., 2007). The initial finding in 2002 was used as an argument for saltationist views of language evolution, i.e., for the claims that language, or at least syntax, emerged suddenly and recently, in all its complexity, as one single (minor) mutation (see e.g., Chomsky, 2010; Berwick and Chomsky, 2011; Piattelli-Palmarini and Uriagereka, 2011). The new findings in 2007 certainly leave room for debate and dialog. For, if one was comfortable using FOXP2 gene to advance saltationist claims prior to the findings in 2007, then one should now certainly be open to the possibility that Neandertals had some form of language, and by extension also our common ancestor. If so, then the question to be addressed is the following: what kind of grammar might have characterized *H. heidelbergensis* and Neandertals? The only way to arrive at specific hypotheses regarding language evolution is to pursue a precise reconstruction based on a linguistic theory and on linguistic variation, and then to subject such hypotheses to interdisciplinary testing.

## What can linguistic theories contribute: reconstructing early stages of grammar

Contributions by linguists are essential to this enterprise, but in order to be helpful, such contributions need to explain clearly, to an interdisciplinary audience, their theoretical postulates and the empirical foundation upon which these postulates rest. The reconstruction proposed in Progovac (2015a, and previous work) is based on the influential framework of Minimalism and its predecessors (e.g., Chomsky, 1995), but it is based only on those postulates that have clear empirical basis and emphasis, and which have survived the test of time and scrutiny^2^. This reconstruction is summarized in this article, but the reader is referred to Progovac (2015a) for the full impact of this proposal, with technical details and the full list of references which provided the background. This reconstruction is adding to the growing body of research advocating a gradualist, incremental approach to the evolution of syntax and language in general, such as Culicover and Jackendoff (2005); Jackendoff (2002); Givón (2009); Gil (2005); Heine and Kuteva (2007); Hurford (2012); Newmeyer (2005); Pinker and Bloom (1990); Progovac (2009); Tallerman (2014), and other work by these and other authors.

I should also point out that Chomsky himself (e.g., Chomsky, 2010; Berwick and Chomsky, 2011) has argued against the very possibility of a gradualist, step-by-step approach to the evolution of syntax, considering that syntax is not decomposable, constituting one complex package which arose through one single sudden event in evolution, such as a minor mutation^3^. Berwick (1998: 338–9) expressed this saltationist view early and eloquently: “there is no possibility of an ‘intermediate’ *syntax* between a non-combinatorial one and full natural language—one either has Merge in all its generative glory, or one has no combinatorial syntax at all” (see also Bickerton, 1990, 1998). My goal is to demonstrate that the basic, foundational postulates of this framework are detachable from the saltationist views. Not only that, but the gradualist approach advocated here actually sheds light on several otherwise puzzling postulates of syntax, including Subjacency, and the small clause beginning of the sentence, as discussed at length in Progovac (2009, 2015a).

In the syntactic framework of Minimalism (and its predecessors), modern sentences and phrases are treated as hierarchical constructs consisting of several layers of structure, built in a binary fashion (see e.g., Adger, 2003 for an excellent overview). The following is the *partial* hierarchy of projections/layers characterizing a typical sentence/clause:^4^

(1) TP > vP > SC/VP

[TP is Tense Phrase (sentence); vP a transitive (higher) verb Phrase; VP the basic Verb Phrase; and SC a Small Clause]^5^.

To put together a sentence such as *Deer will eat fish*, we first assemble the most basic, inner layer, the SC/VP *eat fish* layer. At this point in the derivation, it is still not determined if the fish is going to be the eater (agent) or the eaten (patient), and thus there is arguably no subject/object differentiation (see below)^6^. On the other hand, superimposing the transitivity layer (vP) enables grammaticalized differentiation between agents and patients (as in e.g., *Deer eat fish*). The sentential TP layer, in this case headed by *will*, is then superimposed over the vP layer, to create three layers of syntactic structure. Sentences and phrases in this framework can exhibit additional layers of structure, resulting in highly hierarchical constructs.

Consider how this framework derives transitive (2) and related intransitive (3–4) sentences in English, and how the boundary between them, as well as between what counts as subject vs. object, can get blurred. Importantly, both transitive and intransitive structures start as small clauses which are intransitive, with only one argument (i.e., one participant in the event).

(2) Maria will grow corn.
  - [_SC/VP_ grow corn] →
  - [_vP_ Maria [_SC/VP_ grow corn]] →
  - [_TP_: Maria will [_vP_ Maria [_SC/VP_ grow corn]]]
(3) Corn will grow.
  - [_SC/VP_ grow corn]
  - [_TP_: Corn will [_SC/VP_ grow corn]
(4) Maria will grow.
  - [_SC/VP_ grow Maria]
  - [_TP_: Maria will [_SC/VP_ grow Maria]]

[The cross-out notation indicates the original position in which the subject was merged before moving to TP.]

The theoretical construct in (1) offers a precise method of reconstructing previous syntactic stages in evolution, as formalized in (5). Progovac (2015a) discusses how this method relates to the internal reconstruction method used in historical linguistics.

(5) Structure X is considered to be (evolutionarily) primary relative to Structure Y if X can be composed independently of Y, but Y can only be built upon the foundation of X.

Importantly, the layer upon which the whole sentence rests is the inner, foundational SC layer (*eat fish/grow corn*), which I reconstruct as the initial evolutionary stage of grammar. The logic behind the proposed reconstruction is straightforward: while VP/SC can be composed without a vP or a TP layer, a vP or a TP can only be constructed upon the foundation of a VP/SC. Moreover, while imposing an additional layer of structure upon the foundational SC necessarily results in a hierarchical, layered construct, the SC itself can be a flat, headless, paratactic creation^7^. That is exactly the kind of proto-grammar that this reconstruction arrives at: a flat, tenseless, intransitive, two-slot mold, consisting of just one verb-like and one noun-like element, and in which the subject/object distinction could not be expressed grammatically.

As we start to wonder if this kind of grammar is feasible at all [in the spirit of “what use is half a clause?” (see Progovac, 2008)], consider that we find approximations of such grammars (“living fossils” in the sense of Jackendoff, 1999, 2002) in various constructions in present-day languages^8^. One good example are verb-noun compounds, such as English: *cry-baby, kill-joy, tattle-tale, turn-coat, scatter-brain, tumble-weed, tumble-dung* (insect); Serbian *cepi-dlaka* (lit. split-hair; hair-splitter), *ispi-čutura* (lit. drink-up flask; drunkard), *vrti-guz* (lit. spin-butt; fidget), *jebi-vetar* (lit. screw-wind; charlatan); and Twi (spoken in Ghana) *kukru-bin* (lit. roll-dung; beetle)^9^. These are essentially small clauses created by the two-slot grammar, with just one verb and one noun, without a possibility for any elaboration, or for distinguishing subjects from objects. If we compare e.g., *turn-table* and *turn-coat*, we observe that the former describes a table that turns (table is subject-like), and the latter describes somebody who turns his/her coat, metaphorically speaking (coat is object-like). But, grammatically, these two compounds are identical. Similarly, if we compare *tumble-weed* and *tumble-dung*, we observe that the former describes a weed that tumbles (weed is subject-like), and the latter describes somebody who tumbles the dung (dung is object-like)^10^.

Bickerton (1990, 1998) discusses pidgin languages, as well as early child language, in the light of language evolution, but concludes that these systems are not real language. Bickerton's work has been highly important and influential, especially his insight that in speaking in these simplified ways we can still access the proto-linguistic mode of communication. I also agree with his view that syntax emerged compositionally, by combining words that were available in the one-word stage, as illustrated in detail in Section The Potential and the Limits of Two-Slot Grammars: Shaped by Selection^11^. Where I disagree with Bickerton is in his conclusion that these systems do not constitute real language, and do not have syntax at all. One of the reasons for his stance is his observation that these systems do not obligatorily realize all the arguments of the verb that seem to be obligatory in full adult languages. In other words, there are missing subjects, or objects, or both. However, constructions with arguments gone “missing” are also extremely common in full adult languages. For example, *kill-joy* only includes the object-like noun (cf. “Somebody kills joy”) but there is no expressed subject. In contrast, its hierarchical counterpart *joy-kill-er* (or *dream-squash-er*) expresses both arguments, the subject-like agentive –*er* and the object-like *joy*. Nonetheless, both types of compounds are real language, only reflecting different degrees of syntactic elaboration. Moreover, compounds are by no means the only grammatical structures allowing “missing” arguments. Noun phrases such as *the destruction*, or *the destruction of the bridge*, or *John's destruction of the bridge*, are all completely grammatical and extremely common, even though only the last example has both arguments of the predicate *destruction* saturated, both the destroyer and the destroyed (see also Section What Can Language Variation Tell Us? for a discussion of cross linguistic variation with respect to which arguments can remain unexpressed in different languages).

Not only are the compounds above illustrative of real language, but my approach elevates the processes that put these compounds together to the level of real syntax as well, although a simpler syntax, or “half syntax,” if you will. In order to reconstruct a gradualist, step-by-step approach to the evolution of syntax/language, it is crucial to identify the first, simplest steps, and to show how such first steps brought about communicative advantages over no syntax at all (Section The Potential and the Limits of Two-Slot Grammars: Shaped by Selection), but also how more complex syntax brought about incremental communicative benefits over such first steps (Section What Can Language Variation Tell Us). It is by postulating this simple proto-syntactic stage that one can achieve both, and thus open up the evolution of grammar to gradualist adaptationist accounts.

Moreover, this two-word small (clause) step now provides the foundation upon which more complex hierarchical structures can be built, as illustrated above with the examples (2–4). The small clause, as recognized theoretically as well, continues to provide the necessary scaffolding for building the full sentence, as if the construction of the modern sentence retraces its evolutionary steps. In addition to sentences being built on the foundation of small clauses, there is also cross linguistic (as well as language acquisition) evidence that hierarchical compounds, such as *joy-kill-er* (or *dream-squash-er*) are constructed upon the foundation of flat verb-noun compounds, such as *kill-joy*, as discussed in Progovac (e.g., 2015a).

In this view, human grammar has not been (perfectly) engineered from scratch, in a uniform and exhaustive fashion, but has been tinkered/cobbled together from disparate pieces, starting with simpler grammars and fewer distinctions, and then adding bits and pieces to create a patchwork of structures (Progovac, 2009: 317). Interestingly, the human genome has also been described as “a patchwork quilt …with segments that were picked up at different stages of our ancestry” (Harris, 2015: xvii).

## What can language variation tell us?

The unspecified role of the noun in this reconstructed two-slot grammar (Section What Can Linguistic Theories Contribute: Reconstructing Early Stages of Grammar) can be characterized as the absolutive role, given that such roles are not directly sensitive to the subject/object distinction, or agent/patient distinction. Absolutive-like roles are found not only in languages that are classified as ergative-absolutive (erg-abs), but probably in all languages, in some guise or another, including in the compounds discussed in the previous section. Human languages in fact differ substantially with respect to how they express transitivity, and this reconstructed absolutive-like basis can be seen as the common denominator, the foundation from which the attested variation can arise. The reconstruction offered here is thus synergistic with the findings in linguistic typology, the field concerned with language variation. What makes this synergy possible is the precision of the reconstruction, and the consideration of specific linguistic data.

In erg-abs languages such as Tongan (spoken in Tonga), there is special case marking for an additional, second argument, typically agent or experiencer, and this case marking is called ergative, resulting in structures roughly comparable to: *Eat (by deer*._ERG_) *fish*, where the ergative argument is optional. Intransitive structures comparable to *Eat fish* in Tongan can be vague/unspecified with respect to whether the fish (abs) is eating or being eaten (see also Gil, 2005, for comparable data from Riau Indonesian). In nominative-accusative (nom-acc) languages, such as Serbian, there is special case marking for objects (acc), and here one encounters structures of the kind: (*Deer) eat fish*._ACC_, with the (nominative) subject optionally expressed. In other words, the optional argument in Tongan is the marked, ergative argument, while the optional argument in Serbian is the unmarked, nominative argument. There are also languages which make use of the so-called serial verb constructions, where two small clauses can get strung together to express semantic transitivity, on a par with e.g., *Dog catch, fish eat*, meaning roughly “dog catches it: fish gets eaten”^12^.

Consider first the erg-abs pattern in Tongan (Tchekhoff, 1973: 283):

(6)  Oku       ui         ‘a        Mele.
       _PRES_     call       _ABS_     Mary
       ‘Mary calls.’ / ‘Mary is called.’

(7)  Oku      ui      ‘e        Sione   ‘a        Mele.
       _PRES_    call    _ERG_    John      _ABS_    Mary
       ‘John calls Mary.’

Tchekhoff specifically argues that the grammar of Tongan is sensitive to the first argument vs. second argument distinction, and not to the agent vs. patient distinction, or subject vs. object distinction. Thus, if there is only one argument (whether agent or patient), it will be marked as absolutive, that is, as just first argument (6). The absolutive argument “Mary” in (6) is only specified as a participant involved in the act of calling—hence the possibility of two distinct translations in (6)^13^. However, when the second (ergative) argument is added in (7), this higher argument assumes an agent role, rendering Mary a non-agent, and converging on a single translation in (7). Absolutive structures such as (6) provide an excellent platform from which one can build transitivity, but not in one single uniform way, but in a variety of divergent ways. As recently pointed out by Mufwene (2013, as well as his 2015 LSA Institute lectures in Chicago), cross linguistic differences may reflect different solutions to the same tensions.

Consider next how accusative marking works in Serbian:

(8)  (Petar)       grli      svoj-u        dec-u.
       Peter         hugs    self-_ACC_    children-_ACC_
       ‘Peter hugs/is hugging his children.’

(9)  (Deca)       grle      Petr-a.
       children     hug      Peter-_ACC_
       ‘(The) children hug/are hugging Peter.’

This acc-type grammar is all about object marking, while erg-type grammar is all about marking the higher (second) argument, such as agent. Another difference is that acc marking with transitive verbs is typically obligatory, while erg marking is typically optional. In Serbian-type languages (but not in English) one finds optionality in the expression of the (nominative) subject, as indicated by the use of parentheses in the examples above^14^. But then in Serbian (and other languages) there are ways to avoid the obligatory acc marking, by using a different (non-active) voice, for example the so-called middle voice, which is arguably absolutive-like (Progovac, 2015a,b). Middle voice straddles the boundary between active and passive, transitivity and intransitivity^15^.

(10)  Deca           se     tuku.
         children      _SE_     hit
         ‘Children are hitting each other/somebody else/me.’
         (children as agent)
         ‘One spanks children.’    (children as patient)

Just as is the case with the intransitive absolutive structures in Tongan (6), this middle construction in Serbian leaves its only argument (children) unspecified for semantic role, leading to multiple interpretation possibilities. But in another bizarre twist, this simple absolutive-like pattern now has to be obligatorily flagged with the grammatical word *se*, whose function seems to be just to say: there is no object here (see e.g., Nichols et al., 2004, for what they term “detransitivizing” languages).

Finally, consider an example from Anyi-Sanvi (Kwa family, Niger-Congo) illustrating the serial verb strategy (Van Leynseele, 1975: 191–2):

(11)  cùá       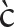
i                    ák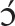           ^!^dì
         dog      catch+_HAB_     chicken     eat
         ‘The dog eats a chicken.’

One can basically see (11) as having two small clauses at its foundation, rather than just one, as postulated for nom-acc patterns in (2–4) above. While each of the small clauses (dog catch; chicken eat) on its own can be seen as intransitive and absolutive-like, when strung together, these clauses express transitive events, by virtue of the event of the first clause being interpreted as causing the event of the second clause^16^.

Interestingly, these structures can express additional information about the event, by virtue of using two verbs. In the example below from Aboh (2009), the first verb (collect) is used not only to introduce the causer/agent (Àsíbá), but also to express the abundance of the action. By varying the verb in the first clause, one can express different aspects of the event.

(12)  Àsíbá     bέ            lέsì       ɖù
         Asiba     collect     rice      eat
        ‘Asiba ate a lot of rice.’

As Aboh (2009) makes it clear, the verb series in today's languages involves various grammatical complexities, and this is also the case with erg-abs and nom-acc patterns.

The significance of the proposed reconstruction is in demonstrating that language variation in the expression of transitivity, even though significant, can be reduced to a single common denominator: the intransitive absolutive-like small clause. The different strategies for transitivity can then be seen as different solutions to the same problem: the problem of having a two-slot grammar, able to fit a verb and only one (first) noun, but desiring to describe an event with more than one participant.

Not only are there reverberations of the foundational absolutive-like pattern in various language types, but many languages in fact have mixed or split systems. For example, so-called “split-ergative” languages are erg-abs with some types of arguments (e.g., those that are less animate on the animacy hierarchy), but nom-acc with others (e.g., those that are more animate). The split often aligns with communicative considerations having to do with the reduction in ambiguity (e.g., Comrie, 1989: 124–137; Aissen, 2003). Comrie (1989: 130) reports that in some languages, such as Hua (spoken in Papua New Guinea), the use of an accusative marker is “conditioned not by any specific rigid cut-off point in the animacy or definiteness hierarchies, but rather … [by] the assessment of likelihood of confusion.” This is where one can see how transitive grammars incur communicative advantages over the postulated flat two-slot stage, providing a rationale for the evolution of such grammars: they can accommodate both subjects and objects, and significantly reduce vagueness in the expression of argument structure.

In fact, the reduced vagueness brought about by hierarchical grammars is what enables the so-called displacement property of language (i.e., the ability to break away from the here-and-now; e.g., Hockett, 1960), considered to be one of the defining properties of human language. This is so because vague (one-word or two-word) proto-structures are much more dependent on the context of the utterance for interpretation, while hierarchical grammars, which can express, with some precision, who does what to whom and when, are much more self-sufficient, and much less reliant on the context/situation of the utterance (Progovac, 2015a)^17^. But how do animacy and context come into play here?

If you are in the two-word stage, and you say *Apple eat* or *Wood chop*, using inanimate nouns, there will not be much room for confusion as to whether the apple is eating or being eaten, or whether the wood is chopping or being chopped. But if you say *Chicken eat*, using an animate noun, there is now a great possibility for confusion. This is why in split languages one is more likely to mark animate patients with acc than inanimate patients. Marking *chicken* with an acc case would disambiguate, rendering it necessarily a patient (object) of eating.

Still, if we utter *Chicken eat* in a specific context, such as the chicken on a plate, there will again not be much possibility for confusion. What will not be possible to do in the proto-syntactic stage like this, however, is to express something that is displaced: *This chicken ate a lot before becoming food itself*. Or something that is completely novel and wild, such as *The apple ate the chicken*. Here the grammar is self-reliant in this respect, and no matter what the context is, or what the common sense tells us, the grammar dictates the interpretation. Such displaced utterances take us away not only from the here-and-now, but also from what makes common sense, what is plausible, expected, and mundane, to what is novel, imagined, wild, and outrageous. For better or for worse, this may well be the most remarkable feature of human language, but this feature is fully enabled only with hierarchical, more elaborated grammars. To appreciate the communicative potential of hierarchical grammars one needs to contrast them with the simpler stage(s).

## The potential and the limits of Two-Slot Grammars: Shaped by Selection

Even though quite simple, the reconstructed two-slot grammar has the ability to combine not only two words, but also two (flat) small clauses, as illustrated in the following AB–AC formulaic “living fossils” from English (13), Twi (spoken in Ghana) (14), and Hmong (spoken in China and northern Southeast Asia) (15). In each example, the clauses are paratactically combined in the sense that they simply stand next to each other, with neither clause being an integral part of the other.^18^

(13)    Monkey see, monkey do.
           First come, first serve.^19^
           Come one, come all.
           Card laid, card played.
           Like father, like son.
           No pain, no gain.
           So far, so good.
           Easy come, easy go.
           Happy wife, happy life.

(14)    a.  Wo      dua,             wo       twa.
                You     sow,             you     reap
           b.  Wo      hwehwea,    wo      hu.
                You     seek             you     find
                make   eat              make   drink

(15)    a.   Ua       noj              ua        haus
                 make  eat              make   drink
                ‘to earn a living’
           b.   Kav    teb              kav      chaw
                 rule    land             rule      place
                 ‘to rule a county’
           c.   Ua      tsov             ua        rog
                 make  tiger            make   war
                 ‘make war’
           d.    Kev   tshaib          kev      nqhis
                  way   hunger        way      thirst
                  ‘famine’
           e.    Cua   daj               cua      dub
                  wind  yellow        wind     black
                  ‘a storm’

Just like flat compounds, as discussed below, these two-by-two formulae can support an abundance of tokens, demonstrating that even these symmetric, flat grammars have an amazing creative and expressive potential^20^. Especially rich and creative with such AB-AC formulae is Hmong (Martha Ratliff, Personal communication). Mortensen (2014) searched a 17 million-word corpus, and found 3253 types of such AB–AC elaborate expressions used by Hmong speakers, and 16,106 tokens^21^. Given the productivity of this strategy in Hmong, it is not possible to dismiss these constructions as just marginal/peripheral creations that get memorized, or creations that are not real language.

A reviewer raises the question of what constitutes a threshold for real language. In my view, as pointed out above, proto-syntax is real language, so much so that it is found in various “fossil” constructions across present-day languages. When we say *killjoy*, or *scarecrow*, or *Easy come, easy go*, I think we would all agree that this is real language, although it shows a much simpler syntax. I consider that the great complexity characterizing modern languages is to a large extent attributable to the long and slow process of accumulation of a medley of different types of constructions which reflect different stages of language evolution. These different constructions have come to specialize for slightly different meanings or functions, making it possible to express a variety of nuanced meanings. Importantly, these ancestral constructions are often seamlessly intertwined with more modern constructions (like a patchwork quilt), in addition to providing a foundation for modern phrases and clauses, making it very hard to say what would constitute the threshold for real language. This is especially true in the light of immense cross linguistic variation in this respect. I hope that probing syntax and syntactic variation deeper by taking into account this evolutionary dimension will help us formulate specific hypotheses regarding this question.

What all human languages and constructions undoubtedly have in common is the binary paratactic platform, that is, the ability to combine two words or two small clauses paratactically, essentially the properties of the reconstructed flat two-slot stage. All the complex hierarchical phenomena, including transitivity and subordination, have alternative routes, as well as precursors, in parataxis (Progovac, 2015a). This is therefore a deep, conservative property of (human) language, the foundation upon which all else rests.

This conclusion also extends to simple combinations of a verb and a noun. A real breakthrough in the expressive abilities would have come at the point when the one-word stage of language, with no syntax, gave rise to the simplest possible syntactic stage: two-word, flat stage, as characterized in Section What Can Linguistic Theories Contribute: Reconstructing Early Stages of Grammar. It is not possible to dismiss verb-noun compounding strategy as not real language, or not useful syntax. In medieval times alone, thousands of such compounds were created (Weekley, 1916), certainly more than nature needs. Such abundance, indeed extravagance, is usually associated with display and sexual selection, the force that also created the peacock's tail.

In order to evaluate the usefulness of this kind of proto-syntax, one needs to contrast it to no syntax at all. The usefulness of something can only be established relative to something else, and this gradualist, incremental approach to the evolution and elaboration of syntax shows exactly that: how each new significant development brings new communicative benefits. The previous section showed how hierarchical (transitive) syntax incurs clear benefits over simple, two-word syntax. Here I show how simple, two-word stage can incur immense communicative benefits over the one-word, non-syntactic stage.

Imagine we are in a population of about 200 hominins who are in the one-word language stage, having command of about 100 proto-words. Suppose next that these are concrete basic vocabulary items, comparable to the lists below; most of these nouns and verbs are taken from the attested verb-noun compounds, anticipating the argument to be made below. This is not meant to be a reconstruction of the proto-lexicon, which would require an independent route, such as looking at the Swadesh lists of words (ranging from 100 to 207). Swadesh words are used by historical linguists to track phonological changes given that they tend to be stable and wide-spread across cultures. It is of potential interest for future work that there is significant overlap between the words on the Swadesh lists and those found inside verb-noun compounds across languages.

(16) Verb-like proto-words
  break, burn, burst, crack, cry, cut, drag, drink, drip, eat, fart, fill, fold, fuck, hang, heck, hunch, kill, lick, lie, peck, pierce, pinch, piss, rattle, rip, roll, run, scatter, scrape, scratch, shake, shit, shove, skew, sing, sit, smoke, spin, spit, split, stink, stroke, suck, sulk, tread, tumble, turn, wag, wipe.
(17) Noun-like proto-words
  ass, baby, back, balls, beard, belly, bird, brain, butt, dung, face, finger, fire, hair, head, heel, leg, mustache, neck, old-woman, penis, shit, skin, sky, snake, sun, tail, throat, vagina, water, wind, wolf, wood.

Among the fossil verb-noun compounds, the ones that specialize for insult predominate (Progovac, 2015a). However, as pointed out by the reviewers, two-word combinations would have had a myriad of other communicative benefits, including in cooperative endeavors, such as hunting, gathering, and child-rearing. Verb-noun compounds do include those that refer to other animals and plants, and are thus not insults (from Progovac, 2015a): *catch-fly* (plant); *cut-finger* (plant); *rattle-snake; shuffle-wing* (bird); *tumble-dung* (insect) (and the equivalent in Twi, *kukru-bin*, beetle); *stink-bug* (and a similar: *smrdi-vrana* [stink-crow] in Serbian; *wag-tail* (bird) [and the equivalent: *verti-hvostka* in Russian; French *pica-flor* (peck-flower, hummingbird) (and a similar *kjuj-drvo* [peck-wood, woodpecker] in Serbian); Tashelhit Berber (spoken in e.g., Morocco) *ssum-sitan* [suck-cow, insect] (and a similar Old English *burst-cow*; insect).

Still, there are two reasons why I focus on insult here. First, I have isolated “living fossils” that can be argued to specialize for insult when referring to humans (Progovac, 2015a). Second, these data reveal a very specific sexual selection scenario, using very specific words and their combinations, which would have led to proto-syntax quite rapidly, as outlined below. Nevertheless, although these particular data and scenario point to sexual selection, we cannot conclude that insult and sexual selection were the main forces in the evolution of language. They may have played only a minor role. Still, identifying, with some evidence, even a small contributor to language evolution is a big step forward.

Even considering insult alone, one is struck by the remarkable increase in expressive abilities brought about by the simplest of syntax. While it would have no doubt been possible to insult with single words (as it is today), in a one-word stage one is severely limited to insults such as: *ass, fart, old-woman, penis, piss, shit, snake, spit, stink, vagina*. Now compare this one-word potential for insult with the possibilities that open up in the two-slot stage (see Progovac and Locke, 2009; Progovac, 2015a for many more colorful examples from a variety of languages).

(18) kill-joy, turn-skin (cf. turn-coat), hunch-back, wag-tail, tattle-tale, scatter-brain, cut-throat, mar-wood (bad carpenter), heck-wood, busy-body, cry-baby, break-back, catch-fly (plant), cut-finger (plant), fill-belly (glutton), lick-spit, pinch-back (miser), shuffle-wing (bird), skin-flint (miser), spit-fire, swish-tail (bird), tangle-foot (whiskey), tumble-dung (insect), crake-bone (crack-bone), shave-tail (shove-tail), wipe-tail, wrynge-tail, fuck-ass, fuck-head, shit-ass, shit-head.
(19) cepi-dlaka ‘split-hair’ (hair-splitter); guli-koža ‘peel-skin’ (who rips you off); vrti-guz ‘spin-butt’ (restless person, fidget); muti-voda ‘muddy-water’ (trouble-maker); jebi-vetar ‘fuck-wind’ (charlatan); vuci-guz ‘drag-butt’ (slow-moving person); gori-guzica ‘burn-butt’ (a person in trouble, burn-breeches); kosi-noga ‘skew-leg’ (person who limps); lezi-baba ‘lie-old-woman’ (loose woman or man); jedi-vek, ‘eat-life’ (one who constantly annoys); podvi-rep ‘fold-tail’ (one who is crestfallen); češi-guz ‘scratch-butt;’ deri-muda ‘rip-balls’ (place name, a steep hill); gladi-kur ‘stroke-dick’ (womanizer); kapi-kur ‘drip-dick’ (name of a slow water spring); liz-guz ‘lick-butt;’ nabi-guz ‘shove-butt;’ piš-kur ‘piss-dick;’ plači-guz ‘cry-butt;’ poj-kurić ‘sing-dick’ (womanizer); seri-vuk ‘shit-wolf.’ (Serbian).

You suddenly have the power to create many novel insults, nasty and witty and often humorous, combinations that have never been heard before. You are able to capture a (complex) trait of a person with only two basic proto-words. Remarkably, even with the verbs and nouns that are common and concrete (16–17) one can create concepts that are quite abstract (18–19). Maybe our ancestors first stumbled upon one or two combinations like this, but then started to actively seek new ones. The point of no return.

According to Progovac and Locke (2009), coining compounds akin to the ones illustrated above would have been an adaptive way to compete for status and sex in ancient times. Their successful use would have enhanced relative status first by derogating existing rivals and placing prospective rivals on notice, and second by demonstrating verbal skill and quick-wittedness. Those individuals who were just a bit better at this game would have left more offspring and thus passed on, generation after generation, the genetic make-up that supports this ability. Darwin (1874) identified two distinct kinds of sexual selection, aggressive rivalry and mate choice, both of which seem relevant for the proposed use of these compounds. This particular scenario provides a clear and rapid path into proto-syntax. In contrast, the scenario invoking cooperative use of such compounds for hunting or gathering purposes does not offer such a clear path.

The vast majority of these compounds are now lost, due to their “unquotable coarseness,” as put in Weekley (see also Mohr, 2013 for a historical perspective on this)^22^. While linguists are typically reluctant to deal with vulgar language of this kind, this type of language may provide indispensable clues into the origins of human language, as well as shed light on continuity with the other species^23^. Based on Darwin (1872), Code (2005: 322) points out that strong emotions expressed in animals are those of lust and hostility, and that they may have been the first verbal threats and intimidations uttered by humans (see also Jay, 1980).

While it is true that human beings today are highly cooperative, this need not have been the case at the point when language was just emerging. It is also true that even today humans can be highly competitive, and to me the two are just two facets of the same coin. We are often ready to harm another being in order to save our own child—that is both competition and cooperation, inextricably intertwined. Language today does seem to depend on trust, as pointed out by a reviewer, but we still also use it for the purposes of insult and deception (in some cases in order to protect or promote a relative or a friend), as well as to compete by displaying one's eloquence with language (relevant for positive selection), and by putting down people who are not as eloquent, or who have a language disorder (relevant for negative selection). These processes of competition and selection must have been even more pronounced and overt in the early linguistic stages. It is also worth pointing out that competing by verbal means is more adaptive than resorting to physical violence. Even if only a fraction of physical fighting in a community was replaced by verbal dueling, this would have ultimately contributed to a better survival of the whole community, but also of the more verbal individuals, at the expense of the more violent ones.

## Genes and geography

Interestingly, in their neurophysiological study of language processing, Bickel et al. (2015) found that listeners have a bias toward interpreting the first unmarked noun phrase in a sentence as an agent, which they take to mean that there is a processing bias/preference for accusative case-marking systems, over ergative case-marking systems. Recall from Section What Can Language Variation Tell Us? that in nom-acc languages agents tend to be in the unmarked (nominative) case form, while in erg-abs languages agents are often (but certainly not always) marked by the ergative case. Bickel et al. also found a statistical skewing in historical change, suggesting that languages are more likely to change toward nom-acc case marking than toward erg-abs case marking. If this is so, then my proposal can provide some rationale for these trends, as well as for the persistence of erg-abs patterns despite such trends. If the absolutive-like intransitive structures are the foundational structures, early to emerge in language evolution, then they not only continue to provide the scaffolding for all other structure building, but they also may involve less effort on the part of the speaker.

The experimental design in Bickel et al.'s (2015) study is from the perspective of the listener, rather than the speaker. Even though the listener may show a preference for accusative marking, as it brings about disambiguation more rapidly, intransitive absolutive structures may require less effort on the part of the speaker. It is also worth pointing out that the additional effort of (acc) object-marking may be an overkill in many conversation settings in which the context provides disambiguation^24^. Aissen (2003) looks at a variety of languages which show what she terms “differential object marking (DOM),” including erg/acc splits, and concludes that DOM is a compromise between two contradictory principles, Iconicity and Economy. For her, Iconicity is at work when overt case marking occurs on an object which can easily be confused for a subject, while Economy simply avoids any case marking.

Importantly, if the above considerations are on the right track, then they lend themselves to interdisciplinary testing. To mention just one possibility, one can search for statistical correlations between these (and other) linguistic parameters and genetic variation across populations. The prediction is that allele frequencies of certain gene(s) (or combinations of genes) in populations will correlate with certain linguistic parameters, given that some haplogroup(s) may provide a slight bias toward learning and processing nom-acc languages, vs. erg-abs languages, on analogy with the proposal for tone in Dediu and Ladd (2007).

We are reminded by Dediu (2015) and Dediu and Ladd (2007) that people are not clones, and that there is widespread inter-individual variation when it comes to language expression and language processing, at least some of which is attributable to genetic factors, through the “many genes with small effects” model (p. 10,944). In the very last footnote of their book, even Berwick and Chomsky (2016: 177) acknowledge that there may indeed exist “some language variation in ‘normal’ human populations that is being uncovered by genome sequencing.” They quote Kos et al. (2012), who found that CNTNAP2 gene SNP variants in human populations affected language processing in otherwise healthy adults. Any such genetic biases can be “very weak at the individual level but get amplified through language use and transmission, such that they influence the trajectory of language change and, ultimately, the distribution of linguistic diversity” (Dediu, 2015: 6). Dediu and Ladd note that this kind of genetic model provides a “solid foundation for gradual, accretionary models of language evolution” (10,947).

Geographically speaking, Bickel et al. (2015) considered a database of 617 languages and concluded that substantial proportions of ergative languages cluster in the Pacific region (New Guinea, Australia, Oceania) and, to a lesser extent, in South America. This skewed geographical distribution can also be used to engage the hypotheses about hominin migrations, as it may be a result of separate waves of migration out of Africa (see also Section Drawing Some Conclusions about the Grammatical Abilities of Our Ancestors: Engaging the Hominin Timeline). While this issue is far from settled, there are some indications from genomic and archeological findings that Southeast Asia was settled earlier than the rest of Eurasia (see e.g., Harris, 2015: 129; 177–78; Gil, 2011; Dediu and Levinson, 2013: 12). Based on sequencing the genome of an Aboriginal Australian man, Rasmussen et al. (2011) conclude that Aboriginal Australians are descendants of an early human dispersal into eastern Asia, possibly 62,000–75,000 years ago, while a separate, later dispersal gave rise to modern Asians, approximately 25,000–38,000 years ago. Consistent with that, and based on a review of genetic, archeological, and environmental data, Petraglia et al. (2010) argue that the expansion into Arabia took place as early as between 70,000 and 130,000 years ago, reaching South Asia by around 78,000 years ago. Their conclusion is that the expansion out of Africa was a complex process, rather than just a single rapid event.

Returning to the discussion of selection, linguists often wonder about how one can distinguish between just historical change vs. genetic evolution. Historical language change is typically considered to have no genetic basis or consequence, while language evolution (and evolution in general) is typically associated with genetic changes and selection. However, these two processes need not be as disjoint as typically seen. As Fitch (2008: 522) observes, “language change does not entail a cessation of selection.” In Progovac (2015a), I look at one concrete, although hypothetical scenario considering a historical change involving tone loss, and conclude that this kind of change may easily be intertwined with a genetic change, given that, after tone loss, the ability for perfect processing of tone will be masked, in the sense of Deacon (2003).

Although both types of change occur, tone loss seems to be more common than tone genesis (e.g., Fitch, 2010: 483, quoting Jespersen, 1922). One salient example of tone loss is Swahili (e.g., Clements and Goldsmith, 1984), which used to have two tonal contrasts, High and Low. Such tonal contrasts are used to distinguish words (or morphemes) which otherwise have the same segments (sounds). There are also typically quite complex rules, differing from language to language, determining if and how tone can detach from its original position and spread to another position. It is relevant in this respect that there is a high degree of variability among second language learners in their ability to discern and learn tone distinctions, which may have a genetic component to it.

Deacon (2003) considers that masking and unmasking of “preadaptations” plays an important role in evolution. As an innovative tool (e.g., language) became more and more essential to successful reproduction, “novel selection pressures unmasked selection on previously ‘neutral’ variants and created advantages for certain classes of mutations that might not otherwise have been favored” (93–94). At the same time, this innovative tool “masked selection on traits made less vital by being supplemented” by the innovative tool, such as perhaps the inventory and specificity of human calls (94). This can also apply to the genesis or loss of tone, or in principle to any other language change. This may also be a good place to be reminded that evolution via natural/sexual selection is not some kind of straightforward progression toward a clearly defined lofty goal, but rather it involves just small and often random local advantages, in competition with a host of other potential advantages. Evolution in this sense is as much about loss as it is about gain. As mentioned in e.g., Harris (2015: 77), humans have lost some of the ancestral immune mechanisms to fight certain diseases, as well as a significant proportion of scent detection abilities.

In this respect, perhaps one more digression is in order. If there indeed was a paratactic stage in language evolution, possibly lasting for a prolonged period of time, then it is likely that our ancestors were genetically selected to be really good and creative with this paratactic language, including with compounding (*cry-baby, spin-butt* for fidget; *scatter-brain*), and with AB–AC patterns (*Easy come, easy go; Wind yellow, wind black*), which may or may not have been accompanied by melodies (Footnote 20). But very few of us living today seem to be still capable of using language in such creative, poetic ways. It could be that by going grammatical, and by becoming slaves to a host of tiny grammatical categories and distinctions, we allowed our other great abilities, including poetic and possibly musical talents, to be masked and thus gradually diminished^25^.

## Genes and neurolinguistics

According to Deacon (2003: 86–87), if language structure arose in a drawn-out coevolutionary process in which both brain and language structures would have exerted selection pressures on one another, then “we should expect to find that human brains exhibit species-unique modifications that tend to ‘fit’ the unique processing demands imposed by language learning and use…”^26^. Whether it will ultimately prove right or wrong, the proposed reconstruction of the evolution of syntax provides several postulates and data sets which are specific enough to allow formulation of concrete hypotheses. Neuroimaging experiments can compare and contrast the processing of flat(ter) proto-syntactic structures, with their more complex hierarchical counterparts. In contrast to the hierarchical counterparts, the flat (fossil) structures are hypothesized to show less focused activation in the Broca's, basal ganglia, and other syntactically relevant networks of the brain, but possibly more activation in other, less linguistically specialized areas. In an ongoing project designed along these lines, we have tested 14 English-speaking and 13 Serbian-speaking subjects, arriving at some significant results, especially involving the Broca's-Basal Ganglia network (Progovac et al., submitted).

Precise and detailed as it is, this linguistic reconstruction is well-positioned to contribute to establishing a larger framework for considering how genetic considerations interact with neurolinguistic considerations in shedding light on language evolution. While linguistic reconstructions can identify ancestral proto-structures, and distinguish them from more recent structures, neuroscience can test if these distinctions are correlated with a different degree and distribution of brain activation, and genetics can shed light on the role of some specific genes in making necessary connections in the brain possible.

To take just one example out of many possibilities, certain experimental findings suggest that the recent FOXP2 mutations are responsible for increased synaptic plasticity and better connectivity among neurons in the brain (for several other genes with comparable effects, see e.g., Dediu, 2015; Hillert, 2015). Using a mouse model, Enard et al. (2009: 968) show that the human version of the FOXP2 gene increases synaptic plasticity and dendrite connectivity. This kind of connectivity contributes to the enhanced capability of cortico-basal ganglia circuits in the human brain that regulate critical aspects of language, cognition, and motor control (Lieberman, 2009).

Enhanced synaptic plasticity and dendrite connectivity may well be what is required for effortless processing of hierarchical, complex syntax. In an fMRI experiment, Liégeois et al. (2003) looked at the processing patterns of KE family, linked to a hereditary language disorder (Specific Language Impairment, SLI), implicating FOXP2 gene. The symptoms of the affected family members (those with the FOXP2 mutation) include difficulties with articulation, as well as the use of simplified morpho-syntax, such as subject drop and the nonsystematic use of plural forms and tense (e.g., Gopnik and Crago, 1991; see also Piattelli-Palmarini and Uriagereka, 2011). This implicates problems with functional categories, including tense and TP. While the unaffected KE family members showed a typical left-dominant distribution of activation involving Broca's areas, the affected members showed a more posterior and more extensively bilateral pattern of activation, as well as significant under-activation in Broca's area and its right homolog.

This may indicate that the affected KE family members are relying more on proto-structures, that is, on more ancient processing strategies. Bringing all these considerations together, very roughly speaking, this approach makes it possible to hypothesize about what kind of brain structure/organization (and genetic make-up) is necessary to support what kind of language, which can in turn lead to cross-fertilization with the findings in archeology and genetics, in an attempt to shed light on hominin language capacities.

## Drawing some conclusions about the grammatical abilities of our ancestors: engaging the hominin timeline

There are certain scenarios for the evolution of grammar/syntax that are inconsistent with the reconstruction introduced here, which means that this reconstruction is at the right level of granularity to engage the questions regarding the hominin timeline. For example, the nature and the degree of crosslinguistic variation in how human languages build upon the foundational paratactic stage (Section What Can Language Variation Tell Us?) suggests that the hierarchical stage did not emerge in all its complexity and in a uniform fashion only once (in Africa), but instead multiple times, and independently, either within Africa, or after the dispersion from Africa. If it had emerged only once, before *H. sapiens* spread out, it would be difficult to explain why there is such profound variation across languages of the world in the expression of transitivity (by ergative, accusative, or other means), or in how they express tense/aspect/mood distinctions, to name just two out of several major parameters of variation.

Under the uniregional hypothesis regarding human origins, this reasoning leads to the conclusion that *H. heidelbergensis*, our common ancestor with Neandertals and Denisovans, did not command hierarchical transitive syntax, but most probably “only” the basic, paratactic, two-slot platform^27^. Neandertals would have, in that case, inherited this paratactic grammar, but could not have inherited hierarchical grammar from *H. heidelbergensis*. But this does not mean, of course, that Neandertals could not, or did not, develop their own kind of hierarchical syntax independently, or perhaps some other kind of language complexity. Given my proposal, this only means that Neandertals did not inherit language with a hierarchical grammar from the common ancestor, and neither did the humans, for that matter.

On the other hand, Neandertals could have stayed with the grammar they inherited from *H. heidelbergensis*, the paratactic two-slot grammar^28^. Even though grammatically simple, this kind of grammar has an amazing potential for expressing a variety of meanings, as illustrated in Section The Potential and the Limits of Two-Slot Grammars: Shaped by Selection. It would have allowed *H. heidelbergensis* and Neandertals, among many other communicative opportunities, to hurl insults at each other in the form of flat compounds (e.g., *cry-baby, cut-throat, scatter-brain, vrti-guz* (spin-butt; fidget), *cepi-dlaka* (split-hair; hair-splitter), *muti-voda* (muddy-water; trouble-maker); to name animals and plants (*rattle-snake, tumble-weed, stink-bug*), as well as to express eternal wisdoms and observations in the form of AB–AC formulae (e.g., *You seek, you find; Like father, like son; Monkey see, monkey do*; *Wind yellow, wind black*). For many more examples, the reader is referred to Progovac (2015a).

Consistent with these considerations, if the paratactic proto-syntax stage already characterized the *H. heidelbergensis* species, this would place the emergence of the flat proto-syntactic stage to at least as far as half million years ago. In fact, my proposal also cannot exclude the possibility that *H. erectus* also had some form of proto-language, especially considering that their brains doubled in size relative to those of the Australopithecus, who lived sometime between 4 and 2 million years ago. There was nothing else at that juncture that would have required as much brain capacity as the early stages of language would have, accompanied by a great increase in expressive abilities and vocabulary size.

For completeness' sake, let me point out that the linguistic considerations explored here, as they stand now, are not capable of choosing between the uniregional and multiregional hypotheses regarding human origins. It has been established that *H. erectus* traveled out of Africa around 1.7 million years ago, spreading to Europe and Asia. According to the much less accepted multiregional hypothesis, the local *H. erectus* populations in Africa, Asia, and Europe differentiated into *H. sapiens* independently, by a process of parallel evolution, as well as due to admixture among the populations (see e.g., Stone and Lurquin, 2007)^29^. If this hypothesis turns out to be correct, or a weak version of it (see e.g., Harris, 2015: 163–164), then, under my approach, it would transpire that *H. erectus*, prior to the migrations out of Africa, already commanded the foundational paratactic grammar, and that the more complex hierarchical grammars emerged separately in different geographical locations, after the dispersion. On this scenario, the hierarchical grammars could have originated much earlier than under the strict uniregional hypothesis, given that the dispersion took place much earlier, around 1.7 million years ago.

There may be another possible scenario for the timeline, which would allow for an earlier timing for hierarchical syntax. Namely, it is possible that hierarchical syntax emerged independently among different populations in Africa, and that, as these different populations migrated to different parts of the world, they brought with them these diverse hierarchical grammars (see also Section Genes and Geography for some discussion). Stringer (2007: 17) mentions the possibility of an African version of multiregionalism.

While it may seem to linguists that it is safer and more prudent to wait this out, and join in only at the point when it is already known for sure how and when hominin migrations took place, and how everything and everybody evolved, it may well be that linguistic input is essential for figuring this out. The only clear way forward is for linguists to formulate testable hypotheses consistent with their areas of expertise, and to allow such hypotheses to be subjected to interdisciplinary scrutiny and testing, even if the chances are that these initial hypotheses will be proven wrong.

## Author contributions

LP is solely responsible for its content.

## Funding

The fMRI project cited in the article was supported by funding to LP from Endowed Distinguished Faculty Fellowship awarded through Wayne State University Humanities Center.

### Conflict of interest statement

The author declares that the research was conducted in the absence of any commercial or financial relationships that could be construed as a potential conflict of interest.

## Footnotes

^1^For a review of Berwick and Chomsky (2016), see Progovac (2016b).

^2^Other linguistic frameworks and theories can lead to convergent results. For example, Heine and Kuteva's (2007) reconstruction based on the framework of grammaticalization renders nouns and verbs as the earliest categories of speech in evolution, reinforcing the reconstruction offered here. Different frameworks illuminate language from different angles.

^3^Additionally, Chomsky (2002) considers natural selection (via tinkering) to be messy and poorly understood: “if you take a look at anything that you don't understand, it's going to look like tinkering” (139). In sharp contrast, Dediu (2015: 131) embraces the “messiness,” and stands in awe of “the incredible complexity and beauty of [genetic influences on speech and languages],…the messiness but also elegance that are expected of products of biological evolution.”

^4^The idea that a sentence (TP) is built upon the foundation of a small clause is one of the most stable and insightful postulates in this syntactic framework (see e.g., Burzio, 1981; Stowell, 1981; Kitagawa, 1985; Koopman and Sportiche, 1991; Chomsky, 1995). “Clause” is a term for a sentence that is not used independently, but is embedded in another sentence (e.g., I saw [*that Marianne left*]). “Small clause” is a term for clauses that have very little syntactic structure internally, certainly not enough to determine what they are headed by (e.g., I saw [*Marianne leave*]; see also Footnote 7).

^5^For the relevance of using both SC and VP as labels for the inner layer, see Progovac (2015a); also Footnote 7.

^6^There are syntactic approaches, associated with Baker's (1988) UTAH hypothesis, which postulate that agents must always and uniformly be generated in the vP layer, and patients in the VP layer. While this hypothesis has provided a useful heuristic device, it has been falsified due to multiple problems even in the analysis of English, but especially in the analysis of absolutive and middle structures across languages (Progovac, 2015b; also 2015a). This is exactly what good hypotheses are supposed to do. The evolutionary approach advocated here can shed light on why there is no strict uniformity in this sense: it is because human grammar has not been perfectly engineered from scratch, but has been tinkered/cobbled together from disparate pieces, as discussed below.

^7^Of note is that the term “small clause” still persists, as there is no agreed-upon head (or center) identified for it. This is in spite of many attempts to render small clauses more in line with hierarchical nature of modern phrases, which are headed (e.g., noun phrases are headed by nouns and tense phrases by tense). The phenomenon of parataxis is discussed and illustrated in Section The Potential and the Limits of Two-Slot Grammars: Shaped by Selection.

^8^In biological literature, “living fossils” are defined as species that have changed little from their fossil ancestors in the distant past, such as, for example, lungfish (Ridley, 1993). Bickerton (1990) and Jackendoff (1999, 2002) introduced the idea of language fossils. In syntax, one can define living fossils as constructions which exhibit rudimentary syntax/semantics, not accounted for by the principles of modern (morpho)syntax, but which nonetheless show some continuity with it. For example, the approximations of this proto-grammar in modern languages serve as necessary foundation for building higher layers of structure, but, when used on their own, they exhibit completely different grammatical behavior, including the lack of Move and recursion, as discussed at length in Progovac (2015a).

^9^I am not claiming that these specific compounds were in use in the proto-syntactic stage, but rather that this kind of two-slot mold was used, the mold into which comparable creations could be poured.

^10^In addition to being illustrative of a most rudimentary grammar, such verb-noun compounds in many languages specialize for derogatory reference and insult when referring to humans, which is of relevance for the sexual selection argument developed in Section The Potential and the Limits of Two-Slot Grammars: Shaped by Selection.

^11^At the same time, I think that holistic and formulaic language is also relevant for language evolution, but perhaps in an indirect way. Today we find a good percentage of language to be formulaic, as pointed out by Wray (e.g., Wray, 2002). It is of interest that such formulaic expressions are typically supported exactly by the reconstructed flat proto-syntax.

^12^See also e.g., Mithun (1991) for additional surprising possibilities that languages exploit to express argument structure (i.e., who does what to whom).

^13^Clearly, there are no “missing” arguments in the structure in (6), as the grammar is such that it allows just one, absolutive argument. It can be elaborated by specifying the second (ergative) argument (7), but this elaboration is not required by the grammar.

^14^It is important to keep in mind that there is a lot of variation, quirk, and complexity within each language type. The division into erg-abs, nom-acc, etc., is in broad strokes only. Even a cursory look at Serbian in this section reveals how much it differs from English, even though both are classified as nom-acc.

^15^If language is engineered from scratch as a perfect system, with well-defined features and categories, then we do not expect to find ambivalent and transitional structures, those that defy the rules and categories posited by that very system. On the other hand, such transitional, intermediate structures are fully compatible with a gradualist evolutionary approach, which invokes tinkering.

^16^Comparable NounVerb—NounVerb structures also arise in the creation of sign languages, such as Nicaraguan Sign Language (e.g., WOMAN PUSH—MAN FALL), as reported in Senghas et al. (1997: 558), and in Al-Sayyid Bedouin Sign Language, as reported in Aronoff et al. (2008).

^17^This is *not* to claim that using language today is not reliant on the context. It is only to say that the introduction of transitivity makes it less reliant in this *one specific* respect, the expression of argument structure.

^18^Correlated with the paratactic nature of their bond is the iconic ordering of these clauses: the event of the first clause is interpreted as preceding and/or causing the event of the second clause.

^19^“First come, first served” is also attested in the corpora, but the one given in the text is more interesting, as it involves an uninflected verb, making it comparable to e.g., “Easy come, easy go” (as opposed to “Easy come, easy gone.”).

^20^Of note is perhaps that such parallel combinations would have been easy to fit onto simple melodies, and to develop musical protolanguage from. In the absence of any grammatical words, such paratactic structures rely on prosodic glue (intonation) to hold them together, and if there was musical protolanguage at any point in hominin evolution (for some recent discussion and references, see Fitch, 2010), then it would have been most useful in these earliest stages of grammar.

^21^At his 2015 LSA Institute plenary address in Chicago, Anthony Woodbury refers to certain exuberant expressions of this kind as *the genius of language*, while Johanna Nichols, at her 2015 LSA Institute plenary address, refers to comparable exuberant expressions as *the point of no return*.

^22^Mohr (2013) provides a documented history of obscene, vulgar language, demonstrating how, in medieval times and beyond, many of the words for body parts and bodily functions were completely appropriate to use. She also discusses the use of such language in writing in public spaces, in Roman times, 2000 years ago, and suggests that such use of vulgar expressions correlates with a much less strict sense of privacy in performing bodily functions, as well as in covering body parts with clothing. Whatever we might think of this kind of language today, it played a much bigger role in the ancient times. Mohr also emphasizes the use of such language to inflict insult upon another, clearly evident as far back as the Roman times, and certainly stretching into the modern era as well.

^23^It has been reported that other primates are capable of very simple two-word combinations, such as *hide peanut* and *hide Kanzi* (see e.g., Greenfield and Savage-Rumbaugh, 1990: 161; regarding bonobo Kanzi). As reported in Patterson and Gordon (1993), the gorilla Koko is not only capable of producing novel compounds, but also of insult, playfulness, and humor. It may be instructive to do an experiment trying to teach other primates some of those “dirty” words and their combinations, and see how motivated they might be to use them.

^24^See e.g., Du Bois (1987) and Newmeyer (2005) for the observation that conversation in discourse tends to involve utterances with only one (fully expressed) argument per verb, where, moreover, the omission of arguments seems to follow an erg-abs pattern.

^25^Those few who are still capable of such artistic expression may be considered as great orators in some cultures, as seems to be the case with skilled Hmong shamans and preachers, whose creative use of lofty AB–AC patterns is highly valued (Martha Ratliff, Personal communication).

^26^According to Darwin (1874: 634), “the largeness of the brain in man, relatively to his body, …may be attributed in chief part to the early use of some simple form of language…”

^27^This would be consistent with the slightly smaller size of the *H. heidelbergensis* brains, in comparison to either humans or Neandertals.

^28^As pointed out by a reviewer, it is possible that the move to hierarchical syntax increased demands on working memory. In this respect, Wynn and Coolidge (2004) propose that working memory may have been enhanced in humans, contributing to the capacity for innovation and experimentation.

^29^Finlayson (2009) points out that the distinctions between *H. habilis, H. erectus, H. sapiens*, and other hominins are not as clear-cut as is typically assumed. Harris (2015: 122) also points to the gradual and mosaic-like nature of human/hominin evolution.
